# Frizzled Receptors as Potential Therapeutic Targets in Human Cancers

**DOI:** 10.3390/ijms19051543

**Published:** 2018-05-22

**Authors:** Chui-Mian Zeng, Zhe Chen, Li Fu

**Affiliations:** 1 Guangdong Key Laboratory for Genome Stability & Disease Prevention, Department of Pharmacology and Carson International Cancer Research Centre, Shenzhen University School of Medicine, Shenzhen 518060, China; 2160220126@email.szu.edu.cn; 2 Zhejiang Key Laboratory of Gastro-Intestinal Pathophysiology, Zhejiang Hospital of Traditional Chinese Medicine, Zhejiang Chinese Medical University, Hangzhou 310006, China; chenzhe@zju.edu.cn

**Keywords:** frizzled receptors, WNT signaling pathway, cancer therapy, antibody, small molecule inhibitor

## Abstract

Frizzled receptors (FZDs) are a family of seven-span transmembrane receptors with hallmarks of G protein-coupled receptors (GPCRs) that serve as receptors for secreted Wingless-type (WNT) ligands in the WNT signaling pathway. Functionally, FZDs play crucial roles in regulating cell polarity, embryonic development, cell proliferation, formation of neural synapses, and many other processes in developing and adult organisms. In this review, we will introduce the basic structural features and review the biological function and mechanism of FZDs in the progression of human cancers, followed by an analysis of clinical relevance and therapeutic potential of FZDs. We will focus on the development of antibody-based and small molecule inhibitor-based therapeutic strategies by targeting FZDs for human cancers.

## 1. Introduction

The frizzled receptors (FZDs) are comprised of ten members (FZD1–FZD10). The common characteristics of these members include an *N*-terminal signal sequence followed by highly conserved cysteine-rich domain (CRD) in extracellular region, a seven-pass transmembrane domain and an intracellular *C*-terminal domain [1]. The WNT proteins, a kind of secreted glycoproteins and ligands of FZDs, are comprised of nineteen members which can bind to the cell surface complex consisted of two elements: a member of the FZD family and the co-receptor Low density lipoprotein receptor related protein 5/6 (LRP5/6). Other heterodimeric receptor complexes comprised of an FZD and the cell surface receptors encoded by the receptor tyrosine kinase like orphan receptor 2 (*ROR2*) and receptor-like tyrosine kinase (*RYK*) genes have also been described [2]. Subsequently, the intracellular *C*-terminal domain of FZDs interacts with the Dishevelled (DVL) protein to transduce the WNT signals to the downstream of the pathway [3]. Each member of FZDs can interact with several different WNT proteins to activate either the canonical WNT/β-catenin or the non-canonical WNT/PCP (planar cell polarity) and WNT/Ca^2+^ signaling pathways [4], thereby activating multiple downstream transcription factors that are important for stem cell regulation, embryonic development, cell polarity, proliferation and differentiation, and especially tumorigenesis [5,6]. To date, the selectivity between individual WNTs and FZDs remains poorly understood. Taking a panoramic view of the WNT signaling pathway, it is easy for us to draw a conclusion that FZDs serve as a core component in both canonical and non-canonical pathways. It is worth mentioning that the co-receptors also play a key role in forming a complex with FZDs for binding WNT ligands and are involved in tumorigenesis. Additionally, hetero-dimerization of FZDs and co-receptors is well-known. Nile et al. recently reported that the CRD of FZDs recognized WNT cis-unsaturated fatty acyl groups which bridged two CRD monomers and mediated FZD receptor dimerization [7]. Earlier, Carron et al. implied that FZD receptor dimerization contributed to transduction of WNT/β-catenin signaling [8].

In the past years, accumulating studies have demonstrated that activation of WNT signaling is crucial for both initiation and progression of human cancers. FZDs function as cell surface receptors of WNT signaling pathway. If FZDs are blocked by antibodies or small molecule inhibitors, the WNT signaling pathway will not be activated so that those downstream target genes (e.g., *CCND1*, *MYC*) will not be activated as well. Therefore, targeting FZDs might be a potential approach for cancer therapy.

In this review, we will shortly introduce the structural features of FZDs and present the current knowledge of biological function, mechanism and clinical relevance of FZDs. We aim to focus on the development of antibody-based and small molecule inhibitor-based therapeutic strategies by targeting FZDs for human cancers.

## 2. Structural Features of Frizzled Receptors

FZDs could be further divided into four subgroups according to their identity. FZD1, FZD2, and FZD7 share about 75% identity with each other, FZD4, FZD9, and FZD10 share 65% identity, FZD5 and FZD8 share 70% identity, FZD3 and FZD6 share 50% amino acid identity. The range of identities shared by receptors from different subgroups is from 20 to 40% [9]. The length of FZDs ranges from 500 to 700 amino acids. The extracellular CRD comprises of 120 to 125 amino residues with ten conserved cysteines and is followed by a hydrophilic linker region which contains 40 to 100 amino residues. The proteins also contain seven hydrophobic domains that are predicted to form transmembrane α-helices. The length of the intracellular *C*-terminal domain is variable and is not very conserved compared with each family member [10].

In addition to the basic structural features of FZDs, the common characteristics between FZDs and other GPCRs include the following: (1) the conserved cysteines in the extracellular loops 1 and 2 that are implicated in the formation of disulfide bonds and (2) a series of charged residues at the *N*- and *C*-terminal of intracellular loop 3 that are indispensable for the coupling of receptor and G protein. However, FZDs lack some domains common to other GPCRs, such as the DRY motif at the *C*-terminal end of the third transmembrane domain [11].

## 3. Biological Functions and Mechanism

The biological functions of FZDs have been elucidated in various cancers and in normal development. A lot of functions that promote cancer are mediated by FZDs including cancer cell proliferation, migration, invasion, angiogenesis, stemness, and chemoresistance after cancer recurrence. Notably, FZDs do not work through a single, well-known WNT signaling pathway but are intertwined with other signaling cascades.

### 3.1. FZD1

It has been reported that FZD1 silencing induced parallel strong decrease in the expression of MDR1 (multidrug resistance) gene with a significant restoration of drug sensitivity in neuroblastoma (NB) cancer cells, which confirmed the FZD1-associated chemoresistance [12]. Another study also demonstrated that interference of FZD1 reversed multidrug resistance in breast cancer cells through the WNT/β-catenin pathway with suppression of MDR1 [13]. Rosiglitazone, a member of the synthetic PPARγ (Peroxisome proliferator-activated receptor γ) ligands, suppressed the multidrug resistance in human ovarian cancer cells by downregulating the expression of FZD1 and MDR1 in a concentration-dependent manner [14]. A recent study has shown that downregulation of FZD1 by miR-135b reversed chemoresistance of non-small cell lung cancer (NSCLC) cells, further confirmed the association between FZD1 and chemoresistance [15]. Collectively, the biological function of FZD1 could be involved in modulating the sensitivity of various human cancers to chemotherapeutic drugs. During normal development, Lapointe et al. indicated that FZD1 is required for normal female fertility and may partly regulate cumulus cell function and oocyte maturation in mice [16].

### 3.2. FZD2 

**Figure 1:**
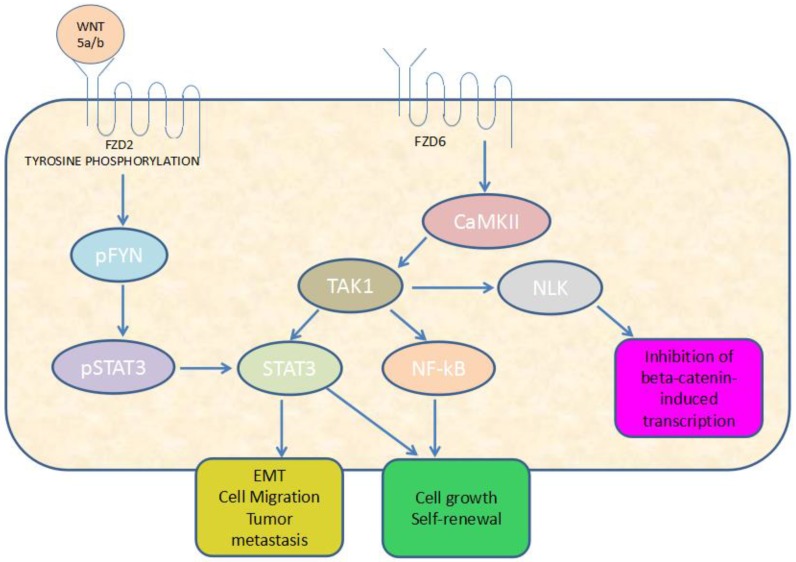
Diagram of a FYN (FYN proto-oncogene)/STAT3 (Signal transducer and activator of transcription 3) signaling for FZD2 in diverse solid tumors and a CaMKII (Ca^2+^/calmodulin-dependent protein kinase II)–TAK1 (TGFβ-activated kinase 1)–NLK (Nemo-like kinase) signaling for FZD6 in mesenchymal-associated phenotype glioblastoma.

Gujral et al. revealed that FZD2 drives epithelial-mesenchymal transition (EMT) and metastasis through a previously unrecognized, non-canonical pathway that included FYN proto-oncogene (FYN), a Src family tyrosine kinase, and signal transducer and activator of transcription 3 (STAT3) transcriptional regulator in diverse solid tumors (Figure 1) [17]. Other studies also suggested that FZD2 contributed to the migration and invasion of human oral squamous cell carcinoma (OSCC) cells, at least partly through regulation of the STAT3 pathway [18]. FZD2 overexpression promoted migration by canonical WNT pathway and facilitated EMT phenotype in endometrial cancer (EC) cells [19]. One study has shown that FZD2 stimulated cell proliferation and promoted cell migration in high-risk NB by regulating β-catenin-dependent and β-catenin-independent signaling pathways. FZD2 blockade by siRNA inhibited NB cell proliferation and xenograft growth, reduced cell motility and induced a less vascularized phenotype [20]. However, one study showed that FZD2 inhibited the cell growth and migration in salivary adenoid cystic carcinomas (SACC), indicating that FZD2 may exert different functions in various cancers [21]. During normal development, Kadzik et al. showed that FZD2 is necessary for domain branch formation during the initial establishment of the respiratory tree and facilitates changes in epithelial cell length and shape in mice. Loss of FZD2 in the lung epithelium leads to the formation of distal cysts in the lung and disrupts the molecular branching program of the lung [22].

### 3.3. FZD3

FZD3 inhibited cell migration and activated cAMP/PKA/Gα(s) signaling pathway by interacting with WNT5a in breast cancer [23]. FZD3 mediated neurite outgrowth in the presence of WNT3a in Ewing tumor cells [24]. During normal development, FZD3 controls axonal polarity and intermediate target entry during striatal pathway development [25] and axonal development in distinct populations of cranial and spinal motor neurons [26]. FZD3-knockout mice show defects in striatal pathway development [25]. FZD3 is a receptor poorly studied among FZD family in human cancers. Previous studies focused on the role of FZD3 in the development of organisms. There is currently no explicit method or antibody that could be taken to target FZD3 for cancer therapy.

### 3.4. FZD4

Interaction of WNT3a with FZD4 activated WNT signaling pathway in acute myeloid leukemia. Functionally, FZD4 expression modulated apoptosis and enhanced WNT3a-induced β-catenin stability in myeloid progenitor cells [27]. Activation of WNT signaling by FZD4 was found in ERG [Erythroblast transformation-specific (ETS)-related gene]-positive prostate cancers. ERG, a member of ETS family of transcription factors, is an oncogene involved in chromosomal translocations and gene fusions. ERG often co-overexpressed with FZD4 in prostate cancer, which led to cancer-promoting phenotypic effects, such as EMT and loss of cell adhesion [28]. FZD4 not only mediated the invasive potential of glioma cells, but also promoted stemness of glioma cells by activating canonical WNT signaling [29]. Downregulation of FZD4 by miR-493 inhibited cell migration in human bladder cancer cells [30]. In cervical carcinoma, miR-505 acted as tumor inhibitor with inverse association with FZD4 [31]. During normal development, Xu et al. revealed that FZD4 is indispensable for vascular development in the retina, and the retinal vasculature of FZD4-knockout mice is defective and abnormal [32].

### 3.5. FZD5

FZD5 increased adhesion to extracellular matrix components including collagen IV, fibronectin and vitronectin in ovarian cancer cells [33]. Inhibition of FZD5/PKC signaling by miR-124 overcame P-glycoprotein-mediated multidrug resistance in renal cell carcinoma [34]. MiR-224 inhibited cell proliferation and migration by targeting FZD5 and inhibiting WNT/β-catenin signaling in breast cancer [35]. Knockout of FZD5 robustly inhibited cell growth in human Ring finger protein 43 (RNF43)-mutant pancreatic ductal adenocarcinoma cells (PDAC) in clonogenic growth assays, and editing of FZD5 significantly inhibited the expression of WNT target genes *AXIN2* and *NKD1*(Naked cuticle homolog 1) [36]. As for the functions of FZD5 in normal development, Burns et al. uncovered that FZD5-mutant mice exhibit abnormal eye development, increased cell death and decreased retinal proliferation by the optic cup stage [37].

### 3.6. FZD6

FZD6-positive NB cells were resistant to doxorubicin and overexpressed mesenchymal markers such as Twist1 and Notch1 [38]. FZD6 also exerted a mediating role in β-carotene-induced uncontrolled proliferation of lung cells [39]. FZD6, targeted by miR-199a-5p and miR-21, played a significant role in the progression of colorectal cancer (CRC) [40]. However, FZD6 suppressed cell proliferation and migration in gastric cancer via the non-canonical WNT pathway [41]. FZD6 regulated fibronectin deposition and assembly of the actin cytoskeleton in MDA-MB-231 cells and metastatic process in vivo, but not proliferation of breast cancer cell lines [42]. One study argued that FZD6 activated CaMKII–TAK1–NLK signaling and inhibited WNT pathway activity through TAK1 while promoting STAT3 and NF-κB signaling (Figure 1) that are important regulators of the mesenchymal-associated phenotype in glioblastoma. Knockdown of endogenous FZD6 significantly decreased sphere formation and cell proliferation of mesenchymal spheres in vitro [43]. As for the functions of FZD6 in normal development, Cui et al. reported that keratin is significantly affected in FZD6-knockout mice. FZD6 is required for the differentiation process of nail development [44].

### 3.7. FZD7

FZD7 plays an important role in stem cell biology and cancer development and progression. Flanagan et al. reported that intestinal epithelium regeneration is impaired in FZD7-knockout mice. FZD7 is indispensable for the maintenance of intestinal epithelial Lgr5+ stem cells and the regeneration of Intestinal Organoids [5], which has been documented recently by Nile et al. by using a small peptide inhibitor dFz7-21 [45]. ΔNp63, an isoform of p63, regulates both normal mammary stem cell activity and tumor initiating activity of breast cancer by increasing FZD7 expression by binding in a conserved active enhancer region of FZD7. And FZD7 knockdown significantly reduced tumor sphere formation in patient derived xenograft tumor [46]. SOX8 and SOX9 induced the FZD7-mediated activation of the WNT/β-catenin pathway to regulate chemoresistance, stem-like properties and EMT in tongue squamous cell carcinoma (TSCC) [47] and hepatocellular carcinoma (HCC) [48]. FZD7 enhanced cell viability and survival, as well as inhibited apoptosis in lung cancer cell lines NCI-H446 and A549 [49]. MiR-142-3p suppressed proliferation and invasion of cervical cells by directly regulating FZD7 expression [50]. FZD7 promoted cell growth, mobility, chemoresistance, metastasis and EMT in esophageal squamous cell carcinoma (ESCC) [51,52]. FZD7 promoted glioma cell proliferation by upregulation of Tafazzin (TAZ) via the β-catenin/TCF-mediated transcription in glioma cells [53]. FZD7 increased ovarian cancer cell proliferation and aggressiveness in vitro via regulation of non-canonical WNT/PCP pathway [54]. Knockdown of FZD7 inhibited metastasis and EMT in cervical cancer cells and gastric cancer cells [55,56], and suppressed tumor transformation in triple negative breast cancer (TNBC) cell lines MDA-MB-231 and BT-20 [57]. FZD7 could also enhance invasion and metastatic capabilities of colon cancer cells through both non-canonical and canonical WNT signaling pathways [58]. Tiwary et al. found that FZD7 related to tumor initiation and metastasis in melanoma cells [59]. It is reported that *Helicobacter pylori* infection promotes FZD7 expression in gastric cancer cells [60]. To this date, FZD7 is the most studied member among FZD family in cancer research.

### 3.8. FZD8

Overexpression of FZD8 promoted, while silencing of FZD8 suppressed prostate cancer cell migration, invasion, stem cell-like phenotypes in vitro and bone metastasis in vivo by the activation of WNT/β-catenin signaling. In addition, wild-type p53 directly interacts with FZD8 promoter transcriptionally repressing FZD8 [61]. It has been reported that FZD8, targeted by long noncoding RNA AK126698, activated by WNT2 ligand and downregulated by miR-100 or miR-520b, could promote cell proliferation, migration, and invasion via activation of WNT/β-catenin pathway in non-small cell lung cancer, spinal osteosarcoma and breast cancer [62,63,64,65]. In addition, FZD8 was found to mediate the interaction of c-Met and WNT/β-catenin signaling, rescue the effects of c-Met inhibition and increase the tumor-initiating ability in cancer stem-like cells of head and neck squamous carcinoma [66]. FZD8 expression was reported to be upregulated after Cisplatin plus TRAIL [Tumor necrosis factor (TNF)-related apoptosis-inducing ligand] treatment in TNBC cells, and inhibition of FZD8 inhibition reduced β-catenin and survivin levels that led to increased apoptosis, indicating that FZD8 plays an important role in drug resistance in TNBC [67]. However, more recent study showed that downregulation of FZD8 expression by K-Ras resulted in a sustained suppression of non-canonical WNT/Ca^2+^ signaling, which led to increased tumorigenicity [68]. In lung cancer, knockdown of FZD8 significantly downregulated the expression of both cyclin D1 and survivin, inhibited cell proliferation and sensitized cell to taxotere treatment in vitro [69].

### 3.9. FZD9

FZD9 expression was reported to be upregulated in astrocytoma [70] and osteosarcoma [71]. FZD9 may relate to angiogenesis in human astrocytoma [70]. FZD9 knockdown inhibited cell proliferation, motility and cyclin D1 expression in HCC and hepatoblastoma cell lines [72].

However, FZD9 was downregulated in acute myeloid leukemia due to the promoter methylation, suggesting it may also function as a tumor suppressor [73]. It has been reported that the direct interaction of WNT7a ligand and its receptor FZD9 repressed cell growth and promoted cell differentiation in NSCLC, indicating an antitumor effect of WNT7a and FZD9 in human cancers [74,75]. Therefore, FZD9 may not be the best target for cancer therapy due to its dual character. During normal development, knockout of FZD9 results in hippocampal and visuospatial learning defects and abnormal B cell development in mice [76,77]. 

### 3.10. FZD10

FZD10 was involved in the progression of synovial sarcoma by regulating actin reorganization and anchorage-independent cell growth [78]. It has been reported that FZD10 is a direct target of SS18-SSX2 which is an oncogenic fusion protein in synovial sarcoma [79]. Nagayama et al. found a strong inverse correlation between FZD10 expression and nuclear β-catenin accumulation in synchronous colorectal tumors, indicating that FZD10 may exert functions via non-canonical WNT signaling pathway [80]. FZD10 overexpression in breast cancer cells due to reduced breast cancer metastasis suppressor 1 like (BRMS1L) level led to aberrant activation of canonical WNT signaling and thus induced EMT and promoted metastasis [81]. Hypoxia-inducible protein-2 (HIG2) was reported to bind to the extracellular domain of FZD10 and activated oncogenic WNT signaling in renal cell carcinoma (RCC) [82].

## 4. Clinical Relevance

There is some evidence that link the overexpression of FZD receptors to poor prognosis in human cancers. Previous studies have demonstrated that FZD receptors were frequently overexpressed in tumor tissues relative to normal tissues. Here, we present the clinical relevance of each FZD member in various cancers (Table 1).

## 5. Therapeutic Potential of FZD Receptors

Disruption of the WNT signaling pathway by blocking FZD receptors provides possibility for rational cancer therapy. Antibody-based and small molecule inhibitor-based therapeutic strategies against the overexpressed FZD receptors could be a translatable approach to overcome cancer. Fukukawa et al. previously reported that FZD10 was significantly transactivated in synovial sarcoma, but not expressed in normal human organs except placenta [85]. Therefore, they generated a murine monoclonal antibody (mAb) named mAb 92-13 that could specifically bind to FZD10 in vitro and vivo, and was internalized into and accumulated in cells expressing FZD10. Moreover, a single intravenous (i.v.) injection of radioisotope-labeled mAb 92-13 strongly suppressed growth of synovial sarcoma in mice without any obvious toxicity [86]. Another study displayed that an anti-FZD7 antibody was able to induce cell death and deplete stem cell properties in FZD7-positve Wilms’ tumor, and reduce in vivo cell proliferation and survival in mouse xenograft model. Moreover, the exogenous expression of two kinds of WNT antagonists, sFRP1 (secreted Frizzled-related protein 1) and DKK1 (Dickkopf WNT signaling pathway inhibitor 1), could promote the sensitivity of Wilms’ tumor cells to the antibody treatment, indicating that a combination of anti-FZDs antibodies and WNT inhibitors may greatly benefit cancer therapy [87]. In addition, an anti-FZD7 antibody with nanoshell was reported to display higher binding affinity for FZD7, increased WNT inhibitory effects and decreased cell viability compared with freely delivered antibody in TNBC cells due to multivalency, which indicated that higher binding affinity is related to higher signaling inhibition and higher therapeutic effects [88]. Nickho and colleagues have isolated single chain variable fragments (scFvs) against FZD7. These scFvs antibodies contain variable domains of heavy and light chains that were linked by an artificial flexible linker, and bind to specific peptide of extracellular domain of FZD7. Functionally, the scFvs inhibited cell growth by inducing cell apoptosis in a TNBC cell line, MDA-MB-231. The reasons why scFvs antibodies are potential therapeutic agents include small size, high affinity, human origin, and specificity [89,90].

OMP-18R5, generated by Gurney et al., was a monoclonal antibody that could interact with FZD1, 2, 5, 7, and 8 by binding to the conserved epitope of extracellular domain of FZD receptors and thereby prevented WNT ligands from binding to FZD receptors, which directly inactivated the canonical WNT pathway. Functionally, OMP-18R5 treatment triggered a reduced growth of human tumor xenografts in mice in several types of human cancers, including breast, pancreatic, colon, and lung tumors [91]. And it was found that combined treatment of OMP-18R5 and several standard-of-care chemotherapeutic agents including Taxol, Irinotecan and Gemcitabine showed striking synergy and significantly reduced cell proliferation and tumorigenicity in both pancreatic and breast tumors [91]. In general, this antibody-based therapeutic strategy by targeting FZDs may be beneficial to the treatment of a broad range of human cancers, and OMP-18R5 currently is in phase I trial [92]. However, one thing to note is that the side effects of OMP-18R5 need to be further evaluated due to its universality and non-specificity.

In a recent study, a small molecule compound SRI37892 was screened to target the transmembrane domain of FZD7 thereby blocking the WNT signal transmission. SRI37892 treatment inhibited LRP6 phosphorylation and downregulated the level of cytosolic free β-catenin, and functionally repressed breast cancer cell viability and colony formation. Therefore, targeting the transmembrane domain of FZD receptors could be a potential therapeutic strategy [93].

Nambotin et al. previously reported that FZD7 was overexpressed in HCC and mediated cancer phenotype. Hence, they have designed small interfering peptides named Rhodamine-tagged heptapeptide protein transduction domain for binding DVL (RHPDs) that were able to disrupt the binding of FZD7 and the PDZ domain of DVL. In vitro, the viability of human HCC cell lines was decreased through apoptosis after RHPDs treatment. In vivo, RHPD-P1, a type of RHPDs, was injected intratumorously in HCC mouse model. It was revealed that RHPD-P1 inhibited tumor growth, which displayed the anti-tumor effect of this small interfering peptide [94]. FJ9, a small molecule inhibitor, was also found to exert the same function as RHPDs to disrupt the interaction between the *C*-terminal tail of FZD7 and the PDZ domain of DVL. FJ9 induced apoptosis in melanoma cell line and non-small cell lung cancer cell line, triggered a reduction of tumor growth in mouse xenograft model [95]. Therefore, small molecule inhibitors that antagonize protein-protein interactions in oncogenic signaling pathways show promise in treating cancer.

dFz7-21, a selective peptide of the FZD7 receptor subclass including FZD1, FZD2, and FZD7, inhibited WNT signaling by binding to the FZD7 CRD subclass at a new site proximal to the lipid-binding groove and altering the dimer interface in turn disrupting the formation of the WNT–FZD–LRP ternary complex. The drastic conformational changes of dFz7-21-FZD7-CRD complex may make the FZD receptor incompetent for ternary complex formation and proper signaling. Although the anti-cancer effect of dFz7-21 has not been demonstrated in this paper, dFz7-21 was able to block Lgr5+ stem cell function, which indicated its potential ability to block FZD7 subclass in cancers [45].

Zachary et al. developed anti-FZD5 antibodies IgG-2919 and IgG-2921. A dose-dependent anti-proliferative effect was observed when treating RNF43-mutant PDAC cell lines HPAF-II, PaTu8988S and AsPC-1 with the FZD5 IgGs, but no obvious effect observed in non-RNF43-mutant PDAC cells. Furthermore, FZD5 antibodies-induced inhibition of RNF43-mutant PDAC cells in vitro was through G0/G1 cell-cycle arrest but not through apoptosis. Experiments in vivo in immunodeficient mice were also performed, 74% and 60% growth inhibition were observed respectively in HPAF-II and AsPC-1 orthotopic tumors treated with 2 mg/kg IgG-2919 compared with control tumors over a 32-days experiment. More importantly, there were no weight loss or visual signs of toxicity. Besides, FZD5 antibodies also inhibited the growth of RNF43-mutant CRC-patient-derived organoids suggesting that FZD5 may be associated with RNF43 mutation across multiple cancer types, expanding the use of FZD5 antibody in cancer treatment [36].

OMP-54F28, a recombinant protein formed by the fusion of the immunoglobulin Fc to the CRD of FZD8, was undergoing clinical trials. OMP-54F28 serves as a decoy receptor competing with FZD8 for WNT ligands. This recombinant protein has been shown to inhibit tumor growth, synergize with chemotherapeutic drugs and decrease frequency of cancer stem cell by blocking the WNT signaling pathway in preclinical models. Recently, a first-in-human phase 1 study of OMP-54F28 was performed in patients with advanced solid tumors. The results showed that the toxicities of OMP-54F28 were mild, and included fatigue, taste alterations, decreased appetite and muscle cramps. Although no notable anti-cancer responses with single OMP-54F28 were observed, the joint treatment of OMP-54F28 and chemotherapeutic drugs may facilitate the anti-cancer activity [96,97,98]. Similarly, soluble FZD7 (sFZD7) created by utilization of the extracellular peptide of FZD7 by Wei et al. also serves as a decoy receptor. sFZD7 was deemed to bind to extracellular WNT ligands, thereby reducing the combination of ligands and FZD7 and inhibiting WNT/β-catenin signaling in HCC. After sFZD7 treatment, the viability of hepatoma cells decreased but normal hepatocytes were uninfluenced, which is the crucial for clinical trials. Moreover, sFZD7 increased the sensitivity of HCC cells to doxorubicin in vitro [99]. And previously, several secreted WNT pathway antagonists have been identified: WNT inhibitor factor (WIF) proteins can interact with WNT ligands; DKK has been reported to bind to LRP5/6; SFRPs, a kind of proteins that share sequence with the CRD of FZDs, compete with FZDs for WNT ligands [100]. From what we have reviewed above, the method of blocking the upstream WNT signal by preventing the binding of a ligand to a receptor looks as effective as directly targeting FZDs.

In addition to FZDs, targeting other components of WNT signaling pathway is not seemingly easy, because they have extra functions. For instance, Adenomatous Polyposis Coli (APC) is not only a part of the destruction complex that limits the level of β-catenin, but also was reported to have a role in directed cell migration [101]. A recent study indicated that APC also contributes to the inhibition of WNT receptor activation independently of WNT ligand [102]. E-cadherin, a protein related to cell adhesion, also can bind to β-catenin to form a complex anchored in cell membrane which ensure the stability of cell-cell junction [103,104]. Thus, if these components are targeted, it’s not just WNT pathway to be influenced. Collectively, FZDs located in cell membrane seem to be more appropriate to be considered as therapeutic targets (Table 2).

## 6. Conclusions and Future Perspectives

**Figure 2:**
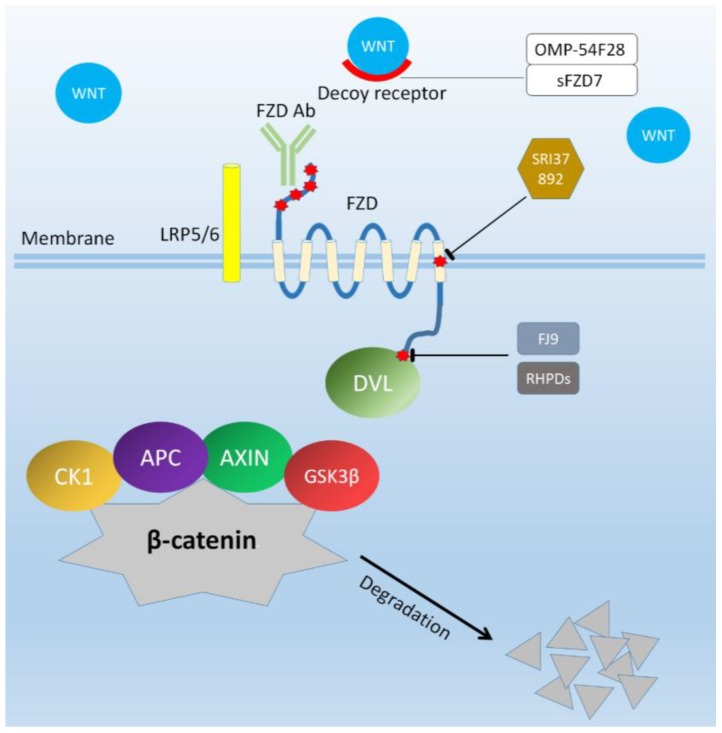
Diagram of WNT (Wingless-type) signaling pathway. OMP-54F28 and sFZD7 act as decoy receptors to reduce the binding of ligands and receptors. The anti-FZD antibody binds to the conserved epitope of extracellular domain of FZDs to form a ligand-receptor complex, preventing the WNT ligands from interacting with FZDs. The small molecule compound SRI37892 targets the transmembrane of FZD7 and block WNT signal transmission. Small interfering peptides RHPDs (Rhodamine-tagged heptapeptide protein transduction domain for binding DVL) and Small molecule inhibitor FJ9 disrupt the joint of FZD7 and DVL. The DVL is not activated so that the WNT signal is unable to be transmitted into the cell. Abbreviations: FZDAb: FZD antibodies; CK1: casein kinase 1; APC: adenomatous polyposis coli; GSK3β: glycogen synthase kinase-3β; DVL: Dishevelled.

WNT signaling pathways have been found to be involved in many important biological processes, especially in tumor development and progression from studies in the past years. As a crucial component of WNT signaling pathway, the FZD family anchored in cell membrane functions in binding to extracellular WNT ligands and transmitting WNT signals to the intracellular molecules, which results in the increased transcription of target genes, many of which encode known oncogenes. In addition, overexpression of FZD family was frequently discovered in various cancers, which mediated cell proliferation, motility, metastasis, stem cell properties, and resistance of tumor cells to chemotherapy. The clinical samples also show the significant relevance between increased expression of FZD family and poor prognosis in a large panel of patients. Therefore, targeting FZD receptors will be a potential and attractive approach for human cancer therapy. Other therapeutic agents specifically targeting the WNT-secretion (Porcupine inhibitors) [105] or β-catenin destruction complex (Tankyrase inhibitors) [106,107] have only recently entered clinical trials and none has yet been approved. Porcupine is a member of O-acyltransferases which specifically palmitoylates WNTs, thereby enabling all WNTs secretion [108]. Tankyrase 1 and tankyrase 2 (TNKS1/2) are part of the larger family of poly (ADP-ribose) polymerase (PARP) enzymes and are the regulators of β-catenin destruction complex. However, the anti-tumor potential of both porcupine inhibitors and tankyrase inhibitors need to be carefully evaluated owing to on-target toxicity (i.e., WNT inhibition and effects on intestinal stem cells, bone turnover, and haematopoiesis) as well as off-target toxicity (i.e., inhibition of other family members of O-acyltransferases or PARP enzymes). In terms of the specificity and selectivity, targeting the signaling cascade at the level of the FZD receptors that are aberrantly overexpressed in tumors may potentiate the therapeutic advantages of WNT-driven cancers. Furthermore, FZD7 expression was reported to be downregulated in all differentiated cell populations relative to undifferentiated human embryonic stem cells, which meant that FZD7 is largely restricted to embryonic tissues with little expression in differentiated tissues [109]. This point makes FZD7 a more attractive target. Currently, several measures have been adopted to target FZD receptors, including the utilization of antibodies, small interfering peptides and small molecule inhibitors (Figure 2). All of these molecules display notable anti-tumor effects in vivo or in vitro. However, it is well-known that some FZDs are still moderately expressed in normal tissues. Therefore, the safety of drugs or antibodies against FZD family need to be further assessed before they are used to treat human cancers. Here, we are looking forward to seeing low or non-toxic and potent anti-FZDs antibody or small molecule inhibitor to be invented in the foreseeable future.

## Figures and Tables

**Table 1:** The clinical relevance of FZDs in different cancers.

Gene	Cancer	Clinical Relevance	Ref.
*FZD1*	Neuroblastoma	Patients with neuroblastoma who relapsed after chemotherapy showed a significant increase of FZD1 expression, but no significant increase was observed in the non-relapsed group of patients.	[12]
*FZD2*	Liver or lung	FZD2 mRNA expression was found to be significantly increased in late stages of primary liver and lung cancers compared with normal tissue and early stage cancer.	[17]
	Endometrial	FZD2 was overexpressed in endometrial cancer tissues compared with the level in normal tissues. Moreover, the expression of FZD2 was positively correlated with markers of mesenchymal cells, such as vimentin and N-cadherin.	[19]
	Salivary adenoid cystic carcinoma	FZD2 expression was downregulated in the samples with metastasis and recurrence when compared to the samples without metastasis.	[21]
*FZD3*	Colorectal	FZD3 protein expression was highly correlated with colorectal carcinogenesis and progression, indicating that it may potentially serve as a prognostic marker.	[83]
*FZD4*	Acute myeloid leukemia	FZD4 protein was expressed in about 80% samples from acute myeloid leukemia patients but rarely expressed in normal bone marrow.	[27]
	Prostate	FZD4 was highly correlated with ERG[Erythroblast transformation-specific (ETS)-related gene] in clinical prostate samples. Higher FZD4 protein expression was often detected in ERG-positive tumors compared with ERG-negative samples.	[28]
	Cervical	FZD4 mRNA expression level in cervical carcinomas was much higher than that in normal cervical tissues in human cervical carcinoma samples.	[31]
*FZD5*	Prostate	FZD5 mRNA expression was significantly higher in the prostate cancer tissues compared with healthy controls.	[84]
*FZD6*	Colorectal	Both FZD6 protein expression and FZD6 mRNA expression were found to be significantly increased compared with adjacent non-tumor samples, which indicated that upregulation of FZD6 correlated with the development of colorectal cancer.	[40]
	Breast	FZD6 expression was significantly associated with reduced distant relapse-free survival in the triple negative breast cancer patient subgroup, but no significant association with other subtypes.	[42]
*FZD7*	Esophageal	FZD7 protein expression was significantly higher in ESCC than that in the adjacent non-tumor tissues. FZD7 overexpression was significantly associated with shorter survival time of patients with ESCC.	[51]
	Glioma	FZD7 protein expression was significantly increased in glioma compared with the adjacent normal tissues.	[53]
	Gastric	FZD7 mRNA expression was found to be significantly upregulated in gastric cancers compared with normal gastric tissues. IHC staining also confirmed the result above and FZD7 overexpression associated with advanced tumor stages and poor survival.	[56]
	Breast	FZD7 expression was significantly higher in the TNBC samples compared with non-TNBC samples.	[57]
	Colorectal	FZD7 mRNA expression examined by real-time PCR was significantly increased in CRC compared with non-tumor tissues. FZD7 overexpression was significantly associated with higher tumor stage.	[58]
*FZD8*	Prostate	Both FZD8 mRNA and protein expression were found to be more notably increased in bone metastatic prostate cancer than in primary prostate cancer and normal prostate tissues.	[61]
	Breast	FZD8 expression was significantly higher in human breast cancer tissues compared with the adjacent normal tissues, and high expression of FZD8 was closely correlated with lymph node metastasis.	[64]
	Lung	Upregulation of FZD8 expression was observed in 85% tumor samples from lung cancer patients when compared to their matched normal lung tissues.	[69]
*FZD9*	Astrocytoma	FZD9 protein was more frequently expressed in malignant astrocytoma than in low-grade astrocytoma.	[70]
	Osteosarcoma	FZD9 expression was higher in osteosarcoma tissues than in the adjacent non-cancerous tissues. The high level expression of FZD9 occurred more frequently in late stage than in the early stage of osteosarcoma.	[71]
*FZD10*	Colorectal	FZD10 mRNA expression was confined to CRC tissues and almost absent from normal mucosa. No IHC staining was observed for FZD10 in normal mucosa, and only tumor cells in polyps and CRC tissues showed spotted staining.	[80]
	Synovial sarcoma	FZD10 mRNA was expressed in synovial sarcoma, but not expressed in normal human organs except placenta. FZD10 protein expression appeared to be absent or low in normal vital organs compared to its highly increased expression in synovial sarcoma tissues.	[85]

**Table 2:** FZD antibodies/inhibitors for various cancers.

Antibodies/Inhibitors	Targets	Cancer Types
mAb 92-13	FZD10	Synovial sarcoma
anti-FZD7 antibody	FZD7	Wilms’ tumor
scFvs	FZD7	Triple negative breast cancer
OMP-18R5	FZD1,2,5,7, and 8	Breast, Pancreatic, Colon and Lung Cancers
SRI37892	FZD7	Breast cancer
RHPDs	FZD7	Hepatocellular carcinoma
FJ9	FZD7	Melanoma, Non-small cell lung cancer
dFz7-21	FZD1, FZD2, FZD7	Unknown
IgG-2919 and IgG-2921	FZD5	RNF43-mutant pancreatic ductal adenocarcinoma
OMP-54F28	Compete with FZD8	Advanced solid tumors
sFZD7	Compete with FZD7	Hepatocellular carcinoma

## Abbreviations

APC	Adenomatous Polyposis Coli
BRMS1L	Breast cancer metastasis suppressor 1 like
CaMKII	Ca^2+^/calmodulin-dependent protein kinase II
CRC	Colorectal cancer
CRD	Cysteine-rich domain
DKK	Dickkopf WNT signaling pathway inhibitor
DVL	Dishevelled
EC	Endometrial cancer
EMT	Epithelial-mesenchymal transition
ERG	ETS-related gene
ESCC	Esophageal squamous cell carcinoma
FZD	Frizzled receptors
GPCRs	G protein-coupled receptor proteins
HCC	Hepatocellular carcinoma
HIG2	Hypoxia-inducible protein-2
LRP5/6	Low density lipoprotein receptor related protein 5/6
NB	Neuroblastoma
NF-kB	Nuclear factor kB
NLK	Nemo-like kinase
NSCLC	Non-small cell lung cancer
PCP	Planar cell polarity
PDAC	Pancreatic ductal adenocarcinoma cells
PPARγ	Peroxisome proliferator-activated receptor γ
ROR2	Receptor tyrosine kinase like orphan receptor 2
RYK	Receptor-like tyrosine kinase
SACC	Salivary adenoid cystic carcinomas
scFvs	Single chain variable fragments
SFRP	Secreted frizzled-related protein
sFZD7	Soluble FZD7
STAT3	Signal transducer and activator of transcription 3
TAK1	TGFβ-activated kinase 1
TAZ	Tafazzin
TNBC	Triple negative breast cancer
TSCC	Tongue squamous cell carcinoma
WIF	WNT inhibitor factor
